# Cortisol, DHEAS, and the cortisol/DHEAS ratio as predictors of epigenetic age acceleration

**DOI:** 10.1007/s10522-025-10307-x

**Published:** 2025-08-16

**Authors:** Rafaela S. C. Takeshita, Amber T. Nguyen, Anthony P. Auger, Wilson C. J. Chung

**Affiliations:** 1 Department of Anthropology, Kent State University, 750 Hilltop Dr, 231 Lowry Hall, Kent, OH USA; 2 Department of Psychology, University of Wisconsin-Madison, Madison, USA; 3 Department of Biological Sciences, Kent State University, Kent, OH USA; 4 School of Biomedical Sciences, Kent State University, Kent, OH USA

**Keywords:** Dehydroepiandrosterone-sulfate, Aging biomarkers, DNA methylation, Stress response, Adrenal hormones, Epigenetic clock

## Abstract

Cortisol has been widely used as biomarker of stress and aging, but confounding effects and disruption of the hypothalamic–pituitary–adrenal axis can lead to misinterpretation of results based on a single measurement. A possible alternative is the co-measurement of cortisol and the adrenal hormone dehydroepiandrosterone-sulfate (DHEAS), a glucocorticoid antagonist that modulates the stress response. Using data from 969 individuals from the Midlife in the United States study, this study aimed to investigate the influence of age, sex, and self-identified biosocial group (SIBG) on DHEAS, cortisol, and the cortisol/DHEAS ratio, to test whether these hormones add predictive power to epigenetic age estimates, and to compare the performance of these three hormonal measures in predicting epigenetic age acceleration (EAA) using sex epigenetic clocks: Horvath, Horvath’s skin & blood (Horvath2), Hannum, PhenoAge, GrimAge, and DunedinPACE. Our findings revealed that age, sex and SIBG significantly influenced all three hormonal measures. Controlling for these biodemographic factors, we found that the cortisol/DHEAS was the best predictor of epigenetic clocks. There was a significant and positive correlation between cortisol and Hannum epigenetic age, and between cortisol/DHEAS ratio in three out of the six clocks (Hannum, Horvath2, PhenoAge), but no significant associations between DHEAS and epigenetic age. The cortisol/DHEAS ratio also had a significant and positive correlation with Hannum EAA. DHEAS and cortisol were not significantly associated with EAA for any epigenetic clock. Our results reinforce the importance of co-measuring cortisol and DHEAS in studies investigating the effect of stress in aging processes.

**Supplementary Information:**

The online version contains supplementary material available at 10.1007/s10522-025-10307-x.

## Introduction

The relationship between stress and aging has attracted increasing research attention due to growing concerns about the effects of chronic stress on longevity. A recent nationwide survey in the United States reported an increase in chronic health conditions and mental illness rates in adults aged 35–44 in 2023 compared to 2019, which have been attributed to increase in overall stress levels due to the COVID-19 pandemic (APA 2023). Long-term stress can also accelerate aging processes through inflammatory processes, cell damage and telomere shortening (Esch et al. 2018; Kaszubowska 2008; Yegorov et al. 2020). With the world population aging rapidly, identifying biomarkers of stress and investigating how they impact aging processes is critical to promote healthy aging and to improve quality of life.

To investigate how stress affects aging processes, scientists have sought to identify biomarkers of biological age, defined as the accumulation of damage in tissue and cells that, over time, will decrease or compromise some physiological functions (Aunan et al. 2016; Gensler and Bernstein 1981; Troen 2003). For instance, blood parameters including c-reactive protein (CRP), interleukin 6 (IL-6), and tumor necrosis factor-α (TNF-α) can indicate an increase in inflammatory processes associated with cell aging (Bortz et al. 2023; de Gonzalo-Calvo et al. 2010). Another well-known biomarker of aging is telomere length, which is based on the fact that telomeres shorten predictably over time, but this process can be accelerated by oxidative stress (Bortz et al. 2023; Diebel and Rockwood 2021; Jylhävä et al. 2017; Zhang et al. 2014). However, these biomarkers have shown limitations as standalone estimators of biological age. For instance, blood-based parameters often need to be interpreted in combination with other health indicators to distinguish aging-related changes from acute inflammation or disease (Consortium et al. 2023). Moreover, inter-individual variability presents a major confounder for telomere length (Aviv et al. 2009), and certain clinical conditions, such as type 2 diabetes, have been associated with a slower rate of telomere shortening (Yang et al. 2025), potentially obscuring true biological aging trajectories.

Recently, epigenetic clocks have emerged as valuable tools for estimating biological age (Horvath and Raj 2018; Levine 2020). The advantage over other biomarkers is that they are based on DNA methylation levels at multiple CpG sites associated with cellular aging, oxidative stress, and inflammatory processes (Horvath and Raj 2018; Levine 2020), therefore relying on a combination of age-related indicators. These clocks have been strongly associated with morbidity and mortality, highlighting their potential as biomarkers of long-term health outcomes (Belsky et al. 2022; Levine et al. 2018; McCrory et al. 2021). Although methylation rates tend to change predictably over time, they can be influenced by lifestyle, social and environmental factors (Abdul et al. 2017; Harvanek et al. 2021; Ryan et al. 2020), as well as by obesity, disease, and stress (Alasaari et al. 2012; Argentieri et al. 2017; Crujeiras and Diaz-Lagares 2016; Kinnally et al. 2011) Notably, several CpG sites included in epigenetic clocks are located within or near glucocorticoid response elements, key regulatory sequences that modulate glucocorticoid activity and the physiological response to stress (Zannas et al. 2015). Because DNA methylation alters gene expression and can exert long-term effects on an individual's stress resilience, epigenetic clocks have been increasingly used to investigate the cumulative biological impact of chronic stress (Boks et al. 2015; Chaix et al. 2017; Vetter et al. 2022).

Cortisol has been widely used as a biomarker of stress due to its role in the stress response and because it can be easily measured from saliva, urine, and blood (El-Farhan et al. 2017; Turpeinen and Hämäläinen 2013). However, the relationship between cortisol and epigenetic age is inconsistent. One study in adolescent girls reported that greater diurnal cortisol levels are associated with accelerated DNA methylation age (Davis et al. 2017). Another study in children from the UK found no significant associations between cortisol and epigenetic age (Tang et al. 2020). One study examined epigenetic age and cortisol trajectories among women that experienced childhood sexual abuse and reported that epigenetic age acceleration was associated with deviations in the age-related pattern of cortisol levels compared to controls (Shenk et al. 2022). These studies indicate that the relationship between epigenetic age and cortisol is complex. Furthermore, studies investigating the relationship between stress hormones and epigenetic age among adults are limited and may be useful for improving our understanding of stress-related aging processes.

Previous studies have highlighted methodological limitations in stress research, including high intra-individual variability of stress biomarkers and poor reliability of single sample measurements in population-based studies (Coste et al. 1994; Golden et al. 2011; Harris et al. 2000; Iqbal et al. 2023; Viardot et al. 2005). Single-point cortisol assessments, in particular, pose challenges in distinguishing acute from chronic stress. Cortisol levels fluctuate in response to immediate stimuli and follow a diurnal rhythm, whereas chronic stress is known to disrupt the hypothalamic–pituitary–adrenal (HPA) axis, often resulting in a blunted cortisol response (Handa and Chung 2019; Herman 2022; Radley and Herman 2022; Sherman et al. 1985). However, obtaining multiple sampling data points can be challenging due to participant compliance. To circumvent this issue, recent studies have used the co-measurement of cortisol and the glucocorticoid antagonist dehydroepiandrosterone-sulfate (DHEAS) to better gauge stress levels (Ahmed et al. 2023; Garner et al. 2011; Gurpinar et al. 2019; Khanfer et al. 2011). DHEAS is the most abundant steroid in human circulation (Hornsby 1995), and compared to the non-sulfate form (DHEA), it is more stable due to a slower clearance rate, which results in the absence of a pronounced circadian rhythm (Longcope 1996). While the full extent of its function is unclear, previous studies demonstrated that DHEAS is involved in the stress response via a glucocorticoid antagonist action (Sacco et al. 2002). In response to a stress stimuli, DHEAS counters the deleterious effects of cortisol in the immune system, brain and metabolism, promoting beneficial effects such as enhanced immune function, neuroprotection, and mood improvement (Bastianetto et al. 1999; Flood and Roberts 1988; Gordon et al. 1988; Hazeldine et al. 2010). However, chronic or repeated stress has been shown to attenuate the DHEAS response (Lennartsson et al. 2013a), leading to a prolonged elevation of circulating cortisol relative to DHEAS. Low DHEAS levels have been associated with depression (Goodyer et al. 1996), chronic pain (Schell et al. 2008), and with increased risk of mortality among men (Ohlsson et al. 2010). Consequently, a high cortisol/DHEAS ratio (Hechter et al. 1997) has been associated with symptoms of chronic stress, including immunodeficiency, depression, anxiety, and cognitive impairment (Boudarene et al. 2002; Carlson et al. 1999; Cruess et al. 1999; Prall et al. 2017). These studies suggest that measuring both cortisol and DHEAS, as opposed to either hormone alone, is a more informative protocol to evaluate health and well-being throughout life (Ritsner et al. 2004; Takeshita et al. 2022; Whitham et al. 2020; Wisniewski et al. 1993).

Importantly, DHEAS levels decline with aging (Belanger et al. 1994; Orentreich et al. 1984a; Šulcová et al. 1997; Young et al. 1999). Low DHEAS levels have been associated with age-related disorders, such as Alzheimer’s disease, cardiovascular disorders, cancer, and immunodeficiency (Arnold et al. 2005; Barrett-Connor et al. 1986; Stamou et al. 2023; Weill-Engerer et al. 2002; Yamaji and Ibayashi 1969; Yanase et al. 1996). For this reason, DHEAS is a promising candidate for evaluating the relationship between stress and aging processes, and previous research has suggested that DHEAS and the cortisol/DHEAS ratio may be useful in predicting biological age more accurately than chronological age (Mazat et al. 2001; Ohlsson et al. 2010; Suh et al. 2023). Yet, to date, there are no studies investigating the relationship between these hormonal measures and epigenetic age.

Beyond aging, DHEAS and cortisol levels are influenced by biodemographic factors, including sex and biosocial group. For instance, DHEAS levels are typically higher in men than in women (Baulieu et al. 2000; Orentreich et al. 1984b; Šulcová et al. 1997), and studies have reported that African Americans exhibit lower DHEAS levels and less steep cortisol declines throughout the day compared to Caucasians (DeSantis et al. 2007; Goldman and Glei 2007; Lee et al. 2021; Morrison et al. 2001). These differences may reflect both biological variation and the influence of social and environmental stressors, and they should be considered when evaluating hormonal effects on aging and health outcomes.

The goal of this study was to test cortisol, DHEAS, and the cortisol/DHEAS ratio as biomarkers of aging using data computed from six epigenetic clocks (Horvath, Horvath’s Skin & Blood clock, Hannum, PhenoAge, GrimAge, DunedinPACE). Our first aim was to examine the influence of biodemographic factors—such as age, sex, and biosocial group—on stress-related hormones. We hypothesized that age, sex and biosocial group would significantly influence all three hormonal measures (cortisol, DHEAS, and the cortisol/DHEAS ratio). Our second aim was to assess whether stress hormones predict epigenetic age, beyond the effects of chronological age. We hypothesized that all three hormone measures would have added predictive value to epigenetic age, when controlling for chronological age. Lastly, our third aim was to compare the predictive performance of the three hormonal measures in explaining variation in epigenetic age acceleration. We hypothesized that the cortisol/DHEAS ratio would be the strongest predictor of epigenetic age acceleration, given its established link to HPA axis imbalance and chronic stress.

## Methods

### Participants

American adult participants in this study were enrolled in the Midlife in the United States (MIDUS) 2 (2004–2006) and MIDUS refresher (2012–2016) Biomarker Project, a national longitudinal study (https://midus.wisc.edu). Participants were asked to identify their sex (male or female) and to respond to an item about ethnic/racial origins using the item “What are your main racial origins—that is, what race or races are your parents, grandparents, and other ancestors?”. However, according to a statement by the American Association of Biological Anthropology, racial categories are not biologically meaningful and do not constitute independent evolutionary lineages (Fuentes et al. 2019). For this reason, we will use the term self-identified biosocial group (SIBG) to refer to these categories hereafter. The possible answers included Caucasian, Black or African American, Native American or Aleutian Islander Eskimo, Asian, Native Hawaiian or Pacific Islander, Multiracial or other.

The following criteria was used for exclusion: (1) SIBG categories with small sample size (< 10%) and (2) participants who did not consent for DNAm data. Caucasian and African American were the two largest SIBG, where 1,387 (75%) of participants identified as Caucasians, and 312 (16.9%) identified as African Americans. It should be noted that many African American participants were recruited from predominantly African American neighborhoods in Milwaukee. A subset of these individuals consented for DNA methylation analysis (N = 969), constituting the final dataset for this study (analytical sample). The population characteristics with the covariates used in our analyses are detailed in Table 1. Considering that stress hormones have been used as indicators of overall health, we did not include any anthropometric variable or data from health questionnaires in our study to investigate the power of hormones in predicting epigenetic age when such data is not available.

**Table 1:** Population demographics of analytical data

SIBG	Age range (mean)	N
Caucasians		727
Men	27–85 (55.9)	351
Women	27–86 (55.2)	376
African Americans		242
Men	32–85 (53)	80
Women	26–82 (51.2)	162

*SIBG* self-identified biosocial group

### Sample collection and assays

For DHEAS measurement, fasting blood samples were collected before 0700 and then processed at the Associated Regional and University Pathologist laboratory (Salt Lake City, UT). Briefly, DHEAS assay was performed with a Roche Modular Analytics E170 analyzer using an Elecsys kit according to manufacturer’s instructions (Roche Diagnostics, Indianapolis, IN). The assay range was between 0.1 and 1000 µg/dL. Limit of detection was 1 µg/dL. The inter-assay coefficient of variation (CV) was 2.9%. The intra-assay CV was between 0.8% and 3.8%.

For cortisol measurement, 12-h ovrnight (1900-0700) urine samples were collected from all participants. Enzymatic Colorimetric Assay and Liquid Chromatography-Tandem Mass Spectrometry was used to measure cortisol and creatinine levels in urine at the Mayo Medical Laboratory (Rochester, MN) as described in previous studies (Taylor et al. 2002). The inter-assay CV was 5.25%. For more details on assay methods, refer to: https://midus-study.github.io/public-documentation/M2P4/Documentation/M2_P4_Blood-Urine-Saliva-Data_Documentation_20240423.pdf

For DNA methylation profiles, whole blood samples following overnight fasting were collected in a BD Vacutainer Tube containing EDTA anti-coagulant and stored at –80 °C. Following DNA extraction, samples were processed for Illumina Methylation EPIC 450 k microarray assays to profile genome-wide methylation. The estimated % methylation at each assayed CpG site was normalized to control for technical sources of variance using the *noob* function in the R *minfi* package. These results were registered onto the list of CpG sites and screened using standard quality DNAm array control metrics. For more details on DNAm analyses, refer to https://midus-study.github.io/public-documentation/Genetics/DNA/M2MR1_Methylation/M2MR1_GEN_DNAmAge_Documentation_20230828.pdf.

### Epigenetic clocks

Previously published algorithms were used to determine various measures of epigenetic age using the methylation data. Specifically, we obtained epigenetic age for Hannum (Hannum et al. 2013), Horvath (Horvath 2013), Horvath’s skin & blood (Horvath2) (Horvath et al. 2018), PhenoAge (Levine et al. 2018), GrimAge (Lu et al. 2019) and DunedinPACE (Belsky et al. 2022). The first five clocks produce estimates of epigenetic age in years, whereas DunedinPACE produces a pace of aging as a multiplying factor. Except for DunedinPACE (r = 0.15), the epigenetic age from all clocks correlated strongly with chronological age (r > 0.87). We calculated EAA for the other five clocks as the residuals from a linear regression model that included chronological age as the independent variable and epigenetic age as the dependent variable. In addition, to investigate shared variance across the different clocks, calculated the mean EAA of the five epigenetic clocks. Horvath and GrimAge EAA were strongly correlated (r = 0.99). EAA from the other clocks had a weak to moderate correlation with one another, with the strongest correlation between Horvath2 and Hannum (r = 0.61), and the weakest correlation between Horvath2 and PhenoAge (r = 0.43).

### Statistical analyses

We used R software version 4.4.2 (R Core Team 2024) for the statistical analyses. In all analyses, we log-transformed the cortisol/DHEAS ratio, instead of the raw values because this transformation is more stable and accurate in quantifying the joint influence of cortisol and DHEAS than the raw ratios (Del Giudice and Gangestad 2022). To examine any potential bias in the final data selected for this study, we compared the mean and frequency of our variables of interest between the subset (analytical sample, N = 969) and the parent data (overall sample, N = 1850). We used t-tests for numerical variables (age and hormonal variables) and Pearson's Chi-squared test for categorical variables (sex and SIBG). There were significant differences in age and SIBG between the analytical and full sample, with the analytical sample being younger and having a higher proportion of African Americans (Table 2). While these differences suggest potential selection bias on age, the analytical sample remains sufficiently large, and the two selected SIBG categories (Caucasians and African Americans) are more balanced, supporting the robustness of this dataset. Therefore, we conducted all statistical analyses using the analytical sample.

**Table 2:** Comparison between demographics and hormonal levels between analytical (N = 969) and overall sample (N = 1850)

Variable	Overall sample	Analytical sample	*p*-value
N	1850	969	
Age [mean (SD)]	55.75 (12.41)	54.60 (12.25)	0.020
Sex = female (%)	1015 (54.9)	538 (55.5)	0.770
SIBG (%)			< 0.001
Caucasian	1387 (75.0)	727 (75.0)	
African American	312 (16.9)	242 (25.0)	
Native American or Aleutian Islander Eskimo	39 (2.1)	0 (0.0)	
Asian	15 (0.8)	0 (0.0)	
Native Hawaiian or Pacific Islander	2 (0.1)	0 (0.0)	
Multiple or other	86 (4.6)	0 (0.0)	
Don’t know	6 (0.3)	0 (0.0)	
Did not answer	3 (0.2)	0 (0.0)	
DHEAS [mean (SD)]	112.64 (84.78)	113.26 (80.65)	0.850
Cortisol [mean (SD)]	3.00 (40.14)	1.61 (1.40)	0.282
Cortisol/DHEAS ratio [mean (SD)]	0.04 (0.53)	0.04 (0.42)	0.727

Data are from the Midlife in the United States

**Fig. 1:**
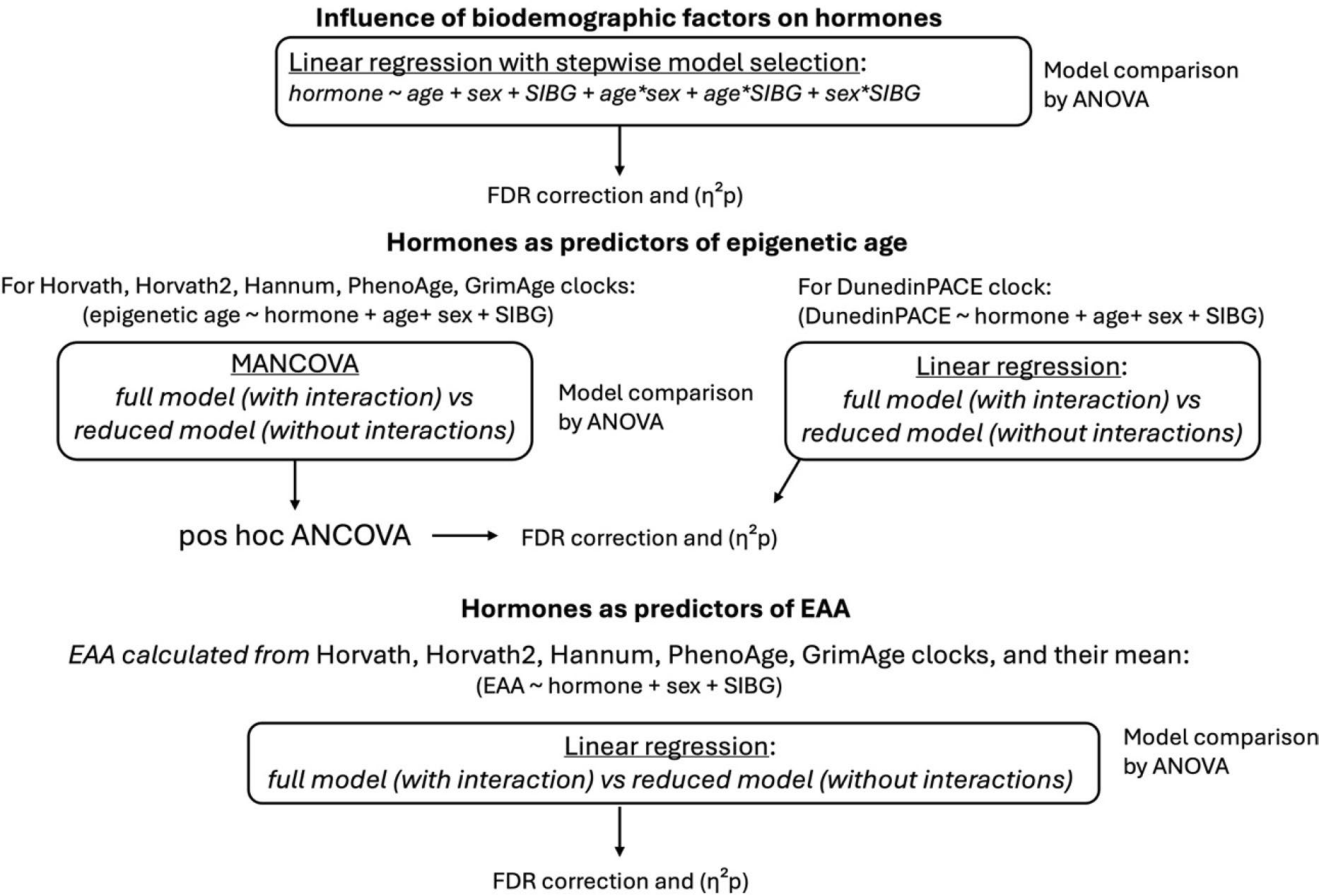
Summary of our three aims and statistical analyses for three hormonal measures (cortisol, DHEAS, cortisol/DHEAS ratio) and six epigenetic clocks (Horvath, Horvath2, Hannum, PhenoAge, GrimAge, DunedinPACE)

The summary of our statistical analyses is illustrated in Fig. 1. First, we used multiple linear regression models to examine the relationship between age, sex and SIBG on each hormonal variable. Before running the models, we tested for multicollinearity by calculating variance inflation factors (VIF) using the *car* package (Belsky et al. 2022). In all models, VIF < 2, which indicates no collinearity issues. We also tested for normality of the residuals using diagnostic plots (histograms of frequency, quantile–quantile plots, and distribution of residuals). Due to non-normality, cortisol and DHEAS were log-transformed, and extreme outliers from all three hormonal measures were removed using the *identify_outliers* function from the *rstatix* package (Kassambara 2019). Normality of the residuals was then confirmed using diagnostic plots. Following Burnham and Anderson (2002), we first built a full model (including all predictors and their interactions), then sequentially removed non-significant interactions. To test whether the removed factors affected the model fit, we compared two sequential models by ANOVA. Factors that significantly affected the model were maintained in the final model. False discovery rate (FDR) *p*-values were adjusted by Benjamini–Hochberg correction. If there were significant interactions in any of the final models, we conducted stratified linear regressions by one of the variables from the interaction, and computed *p*-heterogeneity statistics for the interaction, controlling for interactions between the variables and all other predictors in the model.

Next, we tested hormonal measures as predictors of epigenetic age, while accounting for chronological age. For the five epigenetic clocks that generate epigenetic age (Horvath, Horvath2, Hannum, PhenoAge, GrimAge), we used MANCOVA tests, with epigenetic clocks as the multiple dependent variables, and each hormone in a separate model as the independent variables, with sex, SIBG and chronological age as covariates. DunedinPACE data was not included in the MANCOVA tests because it indicates pace of aging, and not epigenetic age. For this dataset, we used multiple linear regression, with hormonal measures as independent variables, and sex, SIBG and chronological age as covariates. For both MANCOVA and multiple regression models, we first built full models, with interactions between the hormonal measure and the covariates. Then, we removed non-significant interactions in a reduced model and used ANOVA F-tests to confirm that those interactions did not improve the model. We used the same model structure for cortisol, DHEAS and the cortisol/DHEAS ratio to enable model comparison across the different hormonal measures. If MANCOVA tests were significant, we used pos hoc ANCOVA tests for each dependent variable with Benjamini–Hochberg correction for multiple comparisons.

Finally, we investigated which of the hormonal measures can best predict epigenetic age acceleration (EAA). For these analyses, the dataset varied by epigenetic clock (N = 964 to 969) due to the presence of outliers in some of the clocks. For this reason, we used multiple linear regression for each clock and hormone measure separately, with sex and SIBG as covariates. Chronological age was not included in this model because it is already factored out when calculating EAA residuals. We followed the same procedures described above for multiple linear regression, with Benjamini–Hochberg correction for multiple comparisons. Using the function *eta_squared* from the package *effectsize* (Ben-Shachar et al. 2020), we calculated the partial eta-squared (ηp^2^) of each main factor to compare the effect size across different hormonal measures. All plots were generated using the package *ggplot2* (Wickham 2009). A false discovery rate (FDR) *p*-value threshold was set at 0.05.

## Results

### Biodemographic effects on hormones

**Fig. 2:**
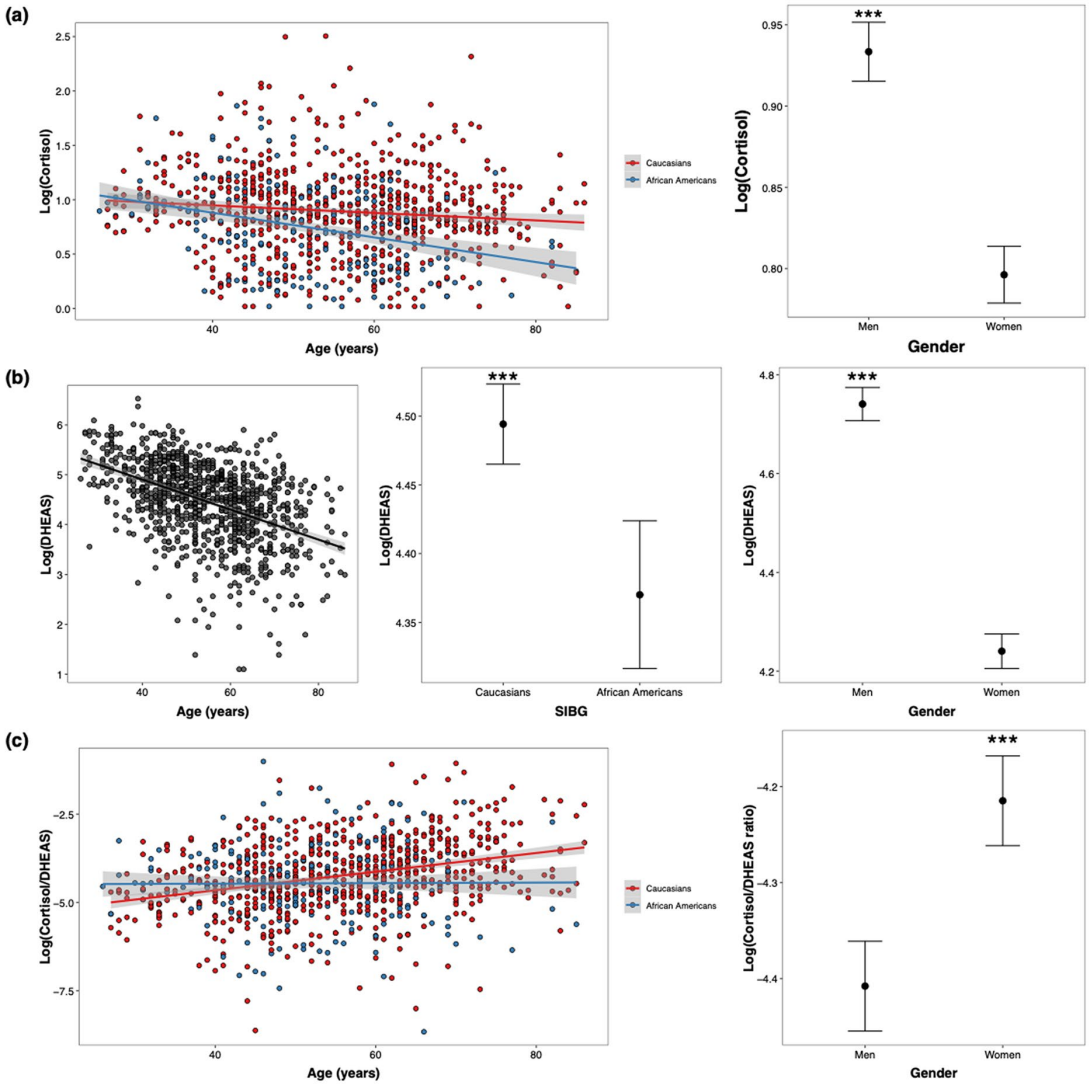
Chronological age, sex and self-identified biosocial group (SIBG) as predictors of **a** cortisol, **b** DHEAS and **c** the cortisol/DHEAS ratio. ***FDR-adjusted *p* < 0.0001

The cortisol model revealed a significant effect of sex, SIBG and age (Table 3), indicating that cortisol levels are lower in women and in African Americans, and it declines with age (Fig. 2a). In addition, there was a significant interaction between SIBG and age. We fit separate linear regression models for each SIBG factor to examine the association between age and cortisol levels. The interaction between age and SIBG was statistically significant (*p*-heterogeneity = 0.0013), indicating that the effect of age differed across the two groups. Among Caucasians, age was associated with a modest decrease in cortisol (β = -0.0036 ± 0.001, *p* = 0.0016, η^2^ = 0.01), whereas in African Americans, the association was stronger (β = -0.0118 ± 0.002, *p* < 0.0001, η^2^ = 0.11).

**Table 3:** Linear regression models to test the effect of age, sex and self-identified biosocial group (SIBG) on cortisol, DHEAS, and the cortisol/DHEAS ratio

Dependent variable	Predictor	β	SE	Partial η^2^	*p-*value (adj.)
Cortisol	Intercept	0.965	0.019	–	–
	Sex (female)	− 0.130	0.025	0.029	< 0.001
	SIBG (African American)	− 0.164	0.029	0.019	< 0.001
	Age	− 0.0036	0.0011	0.026	0.002
	SIBG × age	− 0.0081	0.0025	0.010	0.002
DHEAS	Intercept	4.797	0.0329	–	–
	Sex (women)	− 0.529	0.0427	0.097	< 0.001
	SIBG (African American)	− 0.163	0.0494	0.0007	0.001
	Age	− 0.0322	0.0017	0.238	< 0.001
Log (cort/DHEAS)	Intercept	− 4.395	0.0497	–	–
	Sex (women)	0.237	0.0645	0.0086	< 0.001
	SIBG (African American)	− 0.220	0.0758	0.0104	0.004
	Age	0.0267	0.0029	0.064	< 0.001
	Race × age	− 0.025	0.0065	0.014	< 0.001

Data are from 965 to 967 individuals [outliers (mean ± 1.5 SD) were removed from cortisol (N = 4), DHEAS (N = 2) and the cortisol/DHEAS ratio (N = 2) data] from the Midlife in the United States study

The DHEAS model revealed a significant effect of all predictors, with a negative correlation with age, higher DHEAS levels in Caucasians compared to African Americans, and in men compared to women (Fig. 2b). Age was the stronger predictor, explaining 24% of variation in the data, followed by sex with 9.7%. SIBG had the weakest effect size, explaining less than 0.001% of the variation in the data (Table 3).

The cortisol/DHEAS ratio model had a significant positive correlation with age and a significant effect of sex and SIBG (Table 3), indicating that this hormonal measure increases with age, is lower in African Americans compared to Caucasians, and higher in women compared to men. There was also a significant interaction between age and SIBG, indicating that the aging effect is moderated by SIBG (Fig. 2c). The interaction between age and SIBG was statistically significant (*p*-heterogeneity = 0.0001), indicating that the effect of age differed across the two groups. Among Caucasian participants, age was positively associated with cortisol (β = 0.27 ± 0.03, *p* < 0.0001, η^2^p = 0.11), whereas the association was non-significant among Black participants (β = 0.002 ± 0.01, *p* = 0.8, η^2^p = 0.00).

### Hormones as predictors of epigenetic age

The full MANCOVA models to investigate how hormones perform as predictors of epigenetic age is detailed in Table S1. The model using DHEAS as independent variable had a significant interaction between age and DHEAS. However, this model was not significant different than the reduced model that did not include interactions for any of the hormone measures (Table S2). Therefore, we used the reduced model for MANCOVA to compare the effect size of the three hormonal measures across the epigenetic clocks. We found that all three hormone measures had a significant effect on epigenetic clocks, while controlling for chronological age, sex and SIBG (Table 4). However, pos hoc analyses revealed that DHEAS was not significantly correlated with any of the epigenetic clocks. Cortisol was significantly correlated with Hannum, and the cortisol/DHEAS ratio was significantly correlated with Horvath2, Hannum and PhenoAge clocks (Table 4).

**Table 4:** MANCOVA and pos hoc analyses for DHEAS, Cortisol, and Cortisol/DHEAS as predictors of epigenetic clocks, controlling for age, sex, and SIBG

Hormone	Clock	MANCOVA *p*-value	Partial η^2^ (clock)	*p*-value (FDR adj.)
DHEAS	Horvath	< 0.0001 ***	0.00025	0.624
	Horvath2		0.00264	0.262
	Hannum		0.00208	0.262
	PhenoAge		0.00324	0.262
	GrimAge		0.00030	0.624
Cortisol	Horvath	0.0161 *	0.00106	0.312
	Horvath2		0.00241	0.213
	Hannum		0.01123	0.0049 **
	PhenoAge		0.00553	0.0522
	GrimAge		0.00111	0.312
Log (Cortisol/DHEAS)	Horvath	0.0046 **	0.00067	0.427
	Horvath2		0.00593	0.0365 *
	Hannum		0.01125	0.0048 **
	PhenoAge		0.00544	0.0365 *
	GrimAge		0.00065	0.427

Significant post hoc results are based on FDR-adjusted *p*-values. Data are from 968 individuals [one outlier (mean ± 1.5 SD) was removed from the data] from the Midlife in the United States study

*p < 0.05, **p < 0.01, ***p < 0.001

In addition, we conducted multiple regression models to examine hormones as predictors of DunedinPACE. None of the interactions in the full model was significant for any hormonal measure. Therefore, we used the reduced model (i.e., no interactions) to compare the effect size of the three hormonal measures as predictors of DunedinPACE. We found that none of the hormonal measures was significantly associated with DunedinPACE, after controlling for chronological age, sex and SIBG (Table 5).

**Table 5:** Multiple regression models for DHEAS, Cortisol, and Cortisol/DHEAS as predictors of DunedinPACE, controlling for age, sex, and self-identified biosocial group (SIBG)

Predictors	df	Sum Sq	F value	*p*-value (adj)
(Intercept)	1	331.69	21,270.69	< 0.0001
**DHEAS**	1	0.05	2.90	0.12
Age	1	0.39	25.12	< 0.0001
Sex	1	0.05	3.19	0.1113
SIBG	1	2.95	189.43	< 0.0001
Residuals	963	15.02	–	–
(Intercept)	1	361.54	23,116.57	< 0.0001
**Cortisol**	1	0.00	0.04	0.83
Age	1	0.74	47.53	< 0.0001
Sex	1	0.02	1.46	0.2617
SIBG	1	3.03	193.89	< 0.0001
Residuals	963	15.06	–	–
(Intercept)	1	364.68	23,320.21	< 0.0001
**Log (cortisol/DHEAS ratio)**	1	0.00	0.15	0.74
Age	1	0.68	43.50	< 0.0001
Sex	1	0.03	1.61	0.2553
SIBG	1	3.06	195.68	< 0.0001
Residuals	963	15.06	–	–

Main predictors are highlighted in bold. Data are from 968 individuals [one outlier (mean ± 1.5 SD) was removed from the data] from the Midlife in the United States (MIDUS) study

*SIBG* self-identified biosocial group

### Hormones as predictors of epigenetic age acceleration (EAA)

**Fig. 3:**
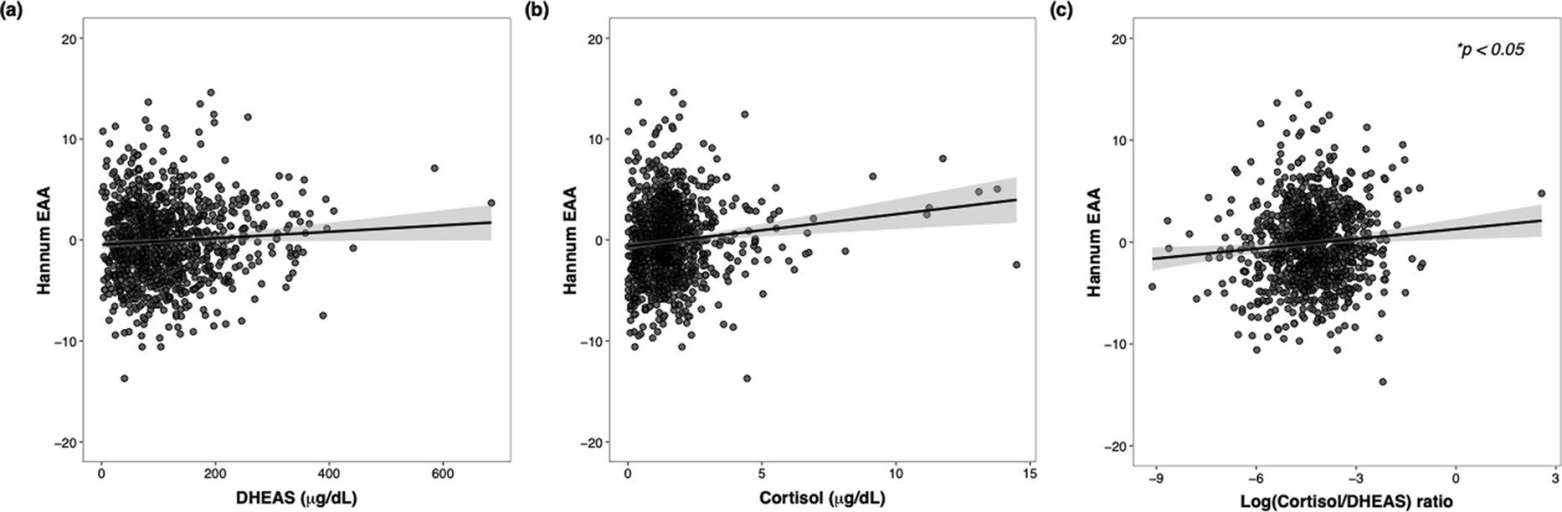
DHEAS (**a**), cortisol (**b**), and the cortisol/DHEAS ratio (**c**) as predictors of Hannum epigenetic age acceleration (EAA). *FDR-adjusted* p* < 0.05

The linear regression models to examine hormones as predictors of EAA included initially each hormone as main factor, sex and SIBG as covariates, as well as interactions between the hormones and these covariates. Given that none of the interactions was significant (Table S3), we built reduced models for each of the three hormonal measures, which include sex and SIBG as covariates. We found that only the cortisol/DHEAS ratio had a significant effect on Hannum EAA, indicating that the cortisol/DHEAS ratio, but not either hormone alone, increases with Hannum EAA (Fig. 3c). There were no significant effects of any hormonal measures for the any other clocks, or their mean (Table 6).

**Table 6:** Multiple regression models for DHEAS, Cortisol, and Cortisol/DHEAS as predictors of epigenetic age acceleration (EAA), controlling for sex, and self-identified biosocial group

Main predictor	β	SE	EAA clock	Partial η^2^	*p-*value (adj)
DHEAS	− 0.0015	0.0018	Horvath	0.000	0.494
	− 0.0022	0.0014	Horvath2	0.001	0.254
	0.0004	0.0016	Hannum	0.005	0.816
	− 0.0039	0.0025	PhenoAge	0.002	0.254
	− 0.0015	0.0018	GrimAge	0.001	0.494
	− 0.0016	0.0012	Mean EAA	0.000	0.327
Cortisol	0.0512	0.0977	Horvath	0.001	0.635
	0.1029	0.0767	Horvath2	0.002	0.324
	0.2442	0.0880	Hannum	0.014	0.050
	0.3364	0.1385	PhenoAge	0.005	0.079
	0.0518	0.0978	GrimAge	0.001	0.635
	0.1277	0.0665	Mean EAA	0.005	0.142
Cortisol/DHEAS ratio	0.1227	0.1279	Horvath	0.000	0.475
	0.2109	0.1003	Horvath2	0.004	0.107
	0.3515	0.1151	Hannum	0.008	0.042 *
	0.4166	0.1814	PhenoAge	0.004	0.079
	0.1215	0.1281	GrimAge	0.000	0.475
	0.2060	0.0871	Mean EAA	0.004	0.079

Significant results are highlighted based on FDR adjusted *p*-values. Data are from 964 to 969 individuals [outliers (mean ± 1.5 SD) were removed from Horvath (N = 2), Horvath 2 (N = 2), Hannum (N = 5), GrimAge, and mean EAA (N = 2) data] from the Midlife in the United States (MIDUS) study

## Discussion

We found that age, sex and SIBG affected all three hormone measures. Cortisol levels were lower in women compared to men, and declined with age, but this effect was stronger in African Americans compared to Caucasians. DHEAS levels declined with age, and they were higher in Caucasians compared to African Americans and in men than in women. The cortisol/DHEAS ratio was higher in women than men and increased with age, but a significant interaction with SIBG indicated that the age effect was significant only among Caucasians. Among the three hormonal measures, the cortisol/DHEAS ratio was the best predictor of epigenetic age. Controlling for age, sex, and SIBG, this hormonal measure had a significant correlation with epigenetic age estimated by Horvath2, Hannum, and PhenoAge clocks. Cortisol had a significant correlation with Hannum clock, and DHEAS was not significantly correlated with any epigenetic clock. In addition, the cortisol/DHEAS ratio was the only hormone significantly associated with EAA, with a positive and significant correlation with Hannum EAA.

The effects of age on DHEAS levels are well established and consistent with our results (Baulieu et al. 2000; Orentreich et al. 1984; Šulcová et al. 1997). The decline of DHEAS levels with aging is associated with the thinning of the adrenal cortex and with age-related disorders, which has prompted its use as biomarker of senescence (Baulieu 1996; Hinson and Raven 1999; Hornsby 1995; Urbanski 2021; Weinstein et al. 1974). While the relationship between age and DHEAS is consistent in the literature, the relationship between age and cortisol is mixed. Some studies show a positive correlation (Oxenkrug et al. 1983; Rotman-Pikielny et al. 2006), while other studies show a negative correlation (Brandtstädter et al. 1991; García-León et al. 2019; Heaney et al. 2012; Sherman et al. 1985), or no significant relationship with age (Halbreich et al. 1984; Oxenkrug et al. 1983; Rotman-Pikielny et al. 2006). This inconsistency may be due to variations in health conditions and lifestyles that influence cortisol levels (Manenschijn et al. 2011; Wosu et al. 2015), suggesting that cortisol is not a reliable predictor of chronological age.

Based on the stronger effect of age on DHEAS compared to cortisol, the increase in cortisol/DHEAS ratio with aging found in this study was expected, and is consistent with the literature (Butcher et al. 2005; Ferrari et al. 2001; Ritsner et al. 2004). High cortisol/DHEAS ratio has been associated with cognitive decline, immunosuppression, hypertension, and metabolic disorders resulting from the imbalance between cortisol and DHEAS (Butcher et al. 2005; Carvalhaes-Neto et al. 2003; De Bruin et al. 2002; Phillips et al. 2010). DHEAS is an important modulator of the stress mechanism that acts via a glucocorticoid antagonist action (Kalimi et al. 1994). During a stress response, DHEAS levels increase to counter the detrimental effects of cortisol, such as immunodepression, neurotoxicity and anxiety (Eser et al. 2006; Hechter et al. 1997; Kimonides et al. 1999; Meza-Rodríguez et al. 2024). Conversely, patients with low DHEAS levels, such as during aging, will have those functions compromised due to the relative high cortisol levels. For this reason, the cortisol/DHEAS ratio has been used as predictor of cognitive decline and health conditions in aging patients (De Bruin et al. 2002; Kalmijn et al. 1998).

We also found that sex influenced all hormones, and SIBG influenced DHEAS and moderated the relationship between cortisol and cortisol/DHEAS ratio with age. Women had lower cortisol and DHEAS levels than men, but the stronger effect of sex on DHEAS resulted in significant higher cortisol/DHEAS ratio in women. The sex difference in DHEAS is consistent in the literature (Orentreich et al. 1984; Šulcová et al. 1997), and has been related to the stimulatory effect of testosterone on DHEAS production in the adrenal gland (Sorwell et al. 2014). In relation to SIBG, the lower DHEAS levels in African Americans could indicate less resilience to stress in this biosocial group, but we found that African Americans had a weaker effect of age on cortisol/DHEAS ratio, which is suggestive of slower aging pace (Cruess et al. 1999). However, this hypothesis differs from several studies reporting evidence of accelerated aging among African Americans (Carter et al. 2019; Levine and Crimmins 2014; Simons et al. 2021; Thorpe et al. 2016). This divergence may suggest that population in our study differs from other African American cohorts, or that the cortisol/DHEAS ratio is not a reliable predictor of chronological age. Based on existing the literature, the latter explanation appears more plausible. Prior studies have shown that cortisol, DHEAS, and the cortisol/DHEA ratio were positively correlated with perceived discrimination (Lee et al. 2021; Ukkola et al. 2001), and one study reported that anxiety symptoms mediated this correlation (Lee et al. 2021). However, previous research has also reported a blunted cortisol awakening response among African Americans, including a study from Milwaukee (Allen et al. 2020), the same population examined in our analyses. This attenuated response, characterized by persistently low cortisol levels at awakening, can be an indicator of dysregulation of the HPA axis (Clow et al. 2010), and has been linked to lifetime and daily experiences of discrimination, as well as to perceived stress (Huynh et al. 2016), low income (Burke et al. 2005) and low education levels (Bennett et al. 2004; Groffen et al. 2015). Furthermore, previous studies have reported lower cortisol awakening response in middle-aged and older African Americans (Samuel et al. 2018; Zilioli 2023), in line with our findings. In the present study, cortisol levels declined with age, with a more pronounced effect observed among African American participants. This finding supports the hypothesis that the cortisol/DHEAS ratio does not vary consistently with chronological age. Consequently, the use of epigenetic clocks to estimate biological age may provide a more informative framework for interpreting the cortisol/DHEAS ratio in the context of stress and aging.

Indeed, when testing hormones as predictors of epigenetic age and EAA, we found that the cortisol/DHEAS ratio was the best predictor. Controlling for chronological age, this hormonal measure had a significant and positive correlation with epigenetic age in three out of the six clocks (Horvath2, Hannum, PhenoAge), compared to one clock for cortisol (Hannum) and none for DHEAS. These results demonstrate that cortisol and the cortisol/DHEAS ratio explain 0.5–1.1% of the variance in the epigenetic data beyond chronological age. Furthermore, the cortisol/DHEAS ratio was the only hormonal measure that had a significant correlation with EAA, and only for the Hannum clock. This result indicates that higher cortisol/DHEAS ratio is associated with faster aging rate, independent of chronological age.

The observed association between epigenetic age acceleration and the cortisol/DHEAS ratio likely reflects the interplay between these two hormones in stress regulation, as well as the adverse physiological effects of chronic stress. Prolonged exposure to stress leads to sustained elevations in cortisol levels. For instance, high hair cortisol concentrations have been reported in endurance athletes (Skoluda et al. 2012), patients with chronic pain (Van Uum et al. 2008), and individuals that experienced major life stress events (Karlén et al. 2011). Persistently elevated cortisol may contribute to age-related diseases due to its detrimental effects in the body, such as neuronal damage in the hippocampus and cortex and immunosuppression (McEwen 1992). These effects are modulated by DHEAS, which exerts anti-glucocorticoid properties and promotes neuroprotection (Maninger et al. 2009; Prall et al. 2017). However, chronic stress exposure has been associated with reduced DHEAS levels. Moriguchi Jeckel et al. 2009 compared salivary DHEA-S levels in female caregivers (considered chronically stressed) and age-matched non-caregivers (considered non-stressed), finding that caregivers had significantly higher stress levels and lower DHEA-S concentrations (Moriguchi Jeckel et al. 2009). Similarly, lower DHEA-S levels have been linked to perceived occupational stress (Lennartsson et al. 2013b). In more advanced stages of chronic stress, the responsiveness of both cortisol and DHEA-S to stressors appears to diminish due to HPA axis dysregulation, characteristic of the “exhaustion” phase of stress (Lennartsson et al. 2022). These shifts in cortisol and DHEAS levels reflect the severity of stress and may explain why the cortisol/DHEAS ratio emerged as the strongest predictor of EAA in our study. Although the effect size of the cortisol/DHEAS ratio on Hannum EAA was modest (0.8%), the results underscore the value of concurrently measuring cortisol and DHEAS when examining the biological impact of stress.

Among the six epigenetic clocks, Hannum had the strongest correlation with hormone measures, followed by Horvath2 and PhenoAge. The Hannum method is based on 71 CpG sites and tracks changes in neutrophils, lymphocytes, monocytes, eosinophils, CD4T/CD8T cell ratio, which associates epigenetic aging with immunosenescence (Hannum et al. 2013). The PhenoAge method, based on 513 CpG sites, has been associated with inflammatory pathways, DNA damage, and changes in transcriptional and translational mechanisms (Levine et al. 2018). The significant association between these two epigenetic clocks and stress hormones may be related to their functions as modulators of the immune system. For example, DHEA, the non-sulfate version of the adrenal steroid, enhances the immune system by preventing cortisol-induced lymphocyte apoptosis (Regelson and Kalimi 1994). Previous studies have shown that DHEA reduces susceptibility to viral infections, while cortisol has the opposite effect (Jacobson et al. 1991). In patients with chronic inflammatory disease, high cortisol and low DHEAS have been associated with increased humoral inflammatory activity (Straub et al. 1998). In this study, Hannum epigenetic age was significantly correlated with cortisol and the cortisol/DHEAS, but only the latter showed a significant association with Hannum EAA. This finding suggests that the actions of the two hormones in the immune system are interdependent.

Another clock significantly correlated with the cortisol/DHEAS ratio was Horvath2. This clock is an improved version of the Horvath method based on 391 CpGs to estimate epigenetic aging for human fibroblasts, keratinocytes, buccal cells, endothelial cells, lymphoblastoid cells, skin, blood, and saliva samples (Horvath et al. 2018). This updated methodology outperformed the original Horvath method, which was not correlated with any hormonal measure. This clock is based on methylation patterns in 353 CpG sites associated with intrinsic tissue variance, such as cell passage number and mutations (Horvath 2013). Therefore, the incorporation of blood markers in the Horvath2 method may have improved the clock as indicative of stress-related aging processes.

Surprisingly, none of the hormonal indexes was significantly correlated with GrimAge, which is based on 12 CpG sites and in plasma proteins adrenomedullin, C-reactive protein, plasminogen activation inhibitor 1, and growth differentiation factor 15, as well as on smoking pack-years as estimator of epigenetic aging (Lu et al. 2019). GrimAge was strongly correlated with Horvath (r = 0.999). The nearly identical correlation between Horvath and GrimAge EAA contrasts with a previous report showing that these two were the least correlated DNAm residuals (Li et al. 2020), but it agrees with a study on firefighters, which suggested that the overlap between CpG sites used to develop the two epigenetic markers may cause this correlation (Jung et al. 2023). Both of them have been correlated with all-cause mortality rate, lung function, cognitive decline and cancer (Chen et al. 2017).

Additionally, none of the hormonal measures was significantly correlated with DunedinPACE, a clock based on 173 CpG sites related to blood biomarkers (Belsky et al. 2022). DunedinPACE measures the pace of aging, while other epigenetic clocks calculate the estimated epigenetic age based on DNAm data (Belsky et al. 2022). Another important difference between DunedinPACE and other clocks is that it was based on longitudinal data over 20 years to track individual differences in aging patterns in multiple organ systems (Belsky et al. 2022). This clock has been associated with morbidity (Belsky et al. 2022), cognitive decline (Savin et al. 2024; Sugden et al. 2022), and brain structural changes (Whitman et al. 2024). Moreover, a healthy lifestyle seems to decelerate aging using this clock (Ke et al. 2024). One possibility for this negative finding is that we did not account for lifestyle. Also, like GrimAge, the DunedinPACE clock accounts for smoking pack-years, which we did not account for (Belsky et al. 2022; Lu et al. 2019). Although further studies that account for those variables are needed to refine our results, our findings demonstrate that stress hormones have potential as biomarkers of stress-related aging processes. Further, our data indicate that Horvath, GrimAge, and DunedinPACE were not associated with adrenal hormones of stress, at least when lifestyle and socioeconomic status were unknown. Therefore, in those cases, Horvath2, Hannum and PhenoAge seem to be better options for evaluating the link between aging and stress hormones.

Our study has some limitations. First, although we found that some clocks were significantly correlated with hormonal measures, the effect size was overall small. These results might possibly improve by including additional data from our population, such as health condition and socioeconomic status. However, considering that cortisol, DHEAS and the cortisol/DHEAS ratio have been widely used as biomarkers of aging and stress, our aim in this study was to test the power of these hormones in predicting aging when such information is not available. A second limitation is that cortisol and DHEAS were measured in different biofluids (urine and blood, respectively), which may influence the accuracy of the cortisol/DHEAS ratio. However, given the relatively short time lag between changes in blood and urinary cortisol (approximately 2 to 4 h) (Fibiger et al. 1984) and the focus on chronic rather than acute stress, this discrepancy is unlikely to have a meaningful impact on our findings. Additionally, the standardization of sample collection times across participants helps to further minimize variability. A third limitation is that our findings may not be fully generalizable to the broader U.S. population. Most African American participants in our dataset were drawn from a single neighborhood in Milwaukee, which may not reflect the diversity of experiences across regions. Additionally, we observed significant age differences between the overall sample and the analytical subsample, which may further constrain the representativeness of our results. Despite these limitations, the study is strengthened by its uniquely large and demographically diverse dataset, which includes representation across sex and SIBG. In addition, our comparative analysis of three hormonal measures and six epigenetic clocks offers novel insights. We demonstrated that while DHEAS showed the strongest correlation with chronological age, the cortisol/DHEAS ratio was a superior predictor of epigenetic age compared to either hormone alone. Furthermore, we identified specific epigenetic clocks that are significantly associated with stress-related hormonal measures, providing important context for interpreting differences across clocks and informing future refinements of epigenetic biomarkers that reflect stress-related mechanisms of aging.

In conclusion, stress hormone levels were significantly influenced by age, sex, and SIBG. After adjusting for these factors, we found that both cortisol and the cortisol/DHEAS ratio were associated with epigenetic age, suggesting that epigenetic clocks enhance the detection of hormone–aging relationships. Among the three hormonal measures, the cortisol/DHEAS ratio emerged as the strongest predictor of both epigenetic age and epigenetic age acceleration (EAA), with the largest effect observed for the Hannum clock, followed by the Horvath2 and PhenoAge clocks. These findings highlight the value of simultaneously measuring cortisol and DHEAS in studies examining the biological impact of stress on aging.

## Supplementary Information

Below is the link to the electronic supplementary material.

Supplementary file1 (DOCX 31 kb)

## Data Availability

All dataset used in this study are publicly available at the Midlife in the United States webpage (https://midus.wisc.edu/)
