# Supplementary material for: Cortisol, DHEAS, and the cortisol/DHEAS ratio as predictors of epigenetic age acceleration
Source: Biogerontology. 2025 Aug 16;26(5):164. doi: 10.1007/s10522-025-10307-x (PMC12357812; doi:10.1007/s10522-025-10307-x)
Supplement: Supplementary file 1 — Supplementary file1 (DOCX 31 kb) [file 10522_2025_10307_MOESM1_ESM.docx]

**Supplementary Tables**

**Table S1.** Full MANCOVA models to test DHEAS, cortisol, and the cortisol/DHEAS ratio as predictors of epigenetic age, with interactions by sex and self-identified biosocial group (SIBG) in 968 individuals from the MIDUS study.

| **Main predictor** | **Term** | **Df** | **Wilks Lambda** | **Approx F** | **Num. Df** | **Den. Df** | ***p*-value** |
| --- | --- | --- | --- | --- | --- | --- | --- |
| DHEAS | age | 1 | 0.074 | 2389.904 | 5 | 956 | <0.0001 |
|  | sex | 1 | 0.902 | 20.722 | 5 | 956 | <0.0001 |
|  | SIBG | 1 | 0.897 | 21.866 | 5 | 956 | <0.0001 |
|  | DHEAS | 1 | 0.992 | 1.546 | 5 | 956 | 0.173 |
|  | age:DHEAS | 1 | 0.982 | 3.599 | 5 | 956 | 0.003 |
|  | sex:DHEAS | 1 | 0.997 | 0.539 | 5 | 956 | 0.747 |
|  | SIBG:DHEAS | 1 | 0.997 | 0.635 | 5 | 956 | 0.673 |
|  | Residuals | 960 |  |  |  |  |  |
| cortisol | age | 1 | 0.075 | 2370.863 | 5 | 956 | <0.0001 |
|  | sex | 1 | 0.902 | 20.730 | 5 | 956 | <0.0001 |
|  | SIBG | 1 | 0.897 | 21.891 | 5 | 956 | <0.0001 |
|  | cortisol | 1 | 0.990 | 1.883 | 5 | 956 | 0.095 |
|  | age:cortisol | 1 | 0.995 | 0.880 | 5 | 956 | 0.494 |
|  | sex:cortisol | 1 | 0.995 | 1.037 | 5 | 956 | 0.395 |
|  | SIBG:cortisol | 1 | 0.994 | 1.184 | 5 | 956 | 0.315 |
|  | Residuals | 960 |  |  |  |  |  |
| Cortisol/DHEAS ratio | age | 1 | 0.074 | 2391.416 | 5 | 956 | <0.0001 |
|  | sex | 1 | 0.902 | 20.752 | 5 | 956 | <0.0001 |
|  | SIBG | 1 | 0.897 | 21.879 | 5 | 956 | <0.0001 |
|  | cortisol/DHEAS ratio | 1 | 0.982 | 3.563 | 5 | 956 | 0.003 |
|  | age:cortisol/DHEAS ratio | 1 | 0.994 | 1.104 | 5 | 956 | 0.357 |
|  | sex:cortisol/DHEAS ratio | 1 | 0.996 | 0.711 | 5 | 956 | 0.615 |
|  | SIBG:cortisol/DHEAS ratio | 1 | 0.997 | 0.598 | 5 | 956 | 0.702 |
|  | Residuals | 960 |  |  |  |  |  |

**Table S2.** Comparison between full (with interactions) and reduced (no interactions) MANCOVA models per hormonal measure as the main predictor, with sex and self-identified biosocial group as covariates.

| Hormone | Residual Df | Df | Generalized Variance | Wilks' Lambda | Approx F | Num Df | *p*-value |
| --- | --- | --- | --- | --- | --- | --- | --- |
| DHEAS | 963 / 960 | -3 | 2.16 / 2.16 | 0.98 | 1.58 | 15 | 0.070 |
| Cortisol | 963 / 960 | -3 | 2.16 / 2.16 | 0.98 | 1.03 | 15 | 0.417 |
| Log(Cortisol/DHEAS) | 963 / 960 | -3 | 2.16 / 2.16 | 0.99 | 0.80 | 15 | 0.675 |

**Table S3.** Full linear models to test cortisol, DHEAS, and the cortisol/DHEAS ratio as predictors of epigenetic age acceleration (EAA) calculated from five epigenetic clocks (Horvath, Horvath2, Hannum, PhenoAge, GrimAge) and their mean, with interactions by sex and self-identified biosocial group (SIBG). Data are from 964-969 individuals from the Midlife in the United States (MIDUS) study.

| **Variable** | **Estimate** | **Standard error** | **Clock** | **Partial η²** | ***p*-value (FDR-adjusted)** |
| --- | --- | --- | --- | --- | --- |
| Cortisol | 0.155 | 0.161 | Horvath | 0.0009 | 0.75 |
| cortisol:sex (women) | -0.111 | 0.204 | Horvath | NA | 0.75 |
| cortisol:SIBG (African American) | -0.174 | 0.249 | Horvath | NA | 0.75 |
| Cortisol | 0.119 | 0.126 | Horvath2 | 0.002 | 0.75 |
| cortisol:SIBG (African American) | 0.188 | 0.196 | Horvath2 | NA | 0.75 |
| cortisol:sex (women) | -0.085 | 0.160 | Horvath2 | NA | 0.75 |
| Cortisol | 0.123 | 0.145 | Hannum | 0.01 | 0.75 |
| cortisol:SIBG (African American) | 0.244 | 0.224 | Hannum | NA | 0.75 |
| cortisol:sex (women) | 0.118 | 0.184 | Hannum | NA | 0.75 |
| Cortisol | 0.271 | 0.228 | PhenoAge | 0.005 | 0.75 |
| cortisol:SIBG (African American) | 0.312 | 0.353 | PhenoAge | NA | 0.75 |
| cortisol:sex (women) | 0.006 | 0.289 | PhenoAge | NA | 0.98 |
| Cortisol | 0.153 | 0.161 | GrimAge | 0.0009 | 0.75 |
| cortisol:sex (women) | -0.108 | 0.204 | GrimAge | NA | 0.75 |
| cortisol:SIBG (African American) | -0.171 | 0.250 | GrimAge | NA | 0.75 |
| Cortisol | 0.138 | 0.110 | Mean EAA | 0.005 | 0.75 |
| cortisol:SIBG (African American) | 0.061 | 0.170 | Mean EAA | NA | 0.82 |
| cortisol:sex (women) | -0.036 | 0.139 | Mean EAA | NA | 0.83 |
| DHEAS | -0.003 | 0.002 | Horvath | 0.0005 | 0.75 |
| DHEAS:sex (women) | 0.006 | 0.004 | Horvath | NA | 0.75 |
| DHEAS:SIBG (African American) | -0.002 | 0.004 | Horvath | NA | 0.75 |
| DHEAS | -0.004 | 0.002 | Horvath2 | 0.001 | 0.75 |
| DHEAS:sex (women) | 0.003 | 0.003 | Horvath2 | NA | 0.75 |
| DHEAS:SIBG (African American) | 0.002 | 0.003 | Horvath2 | NA | 0.75 |
| DHEAS:SIBG (African American) | -0.003 | 0.004 | Hannum | NA | 0.75 |
| DHEAS:sex (women) | 0.002 | 0.003 | Hannum | NA | 0.75 |
| DHEAS | 0.001 | 0.002 | Hannum | 0.005 | 0.83 |
| DHEAS | -0.005 | 0.003 | PhenoAge | 0.002 | 0.75 |
| DHEAS:sex (women) | 0.007 | 0.005 | PhenoAge | NA | 0.75 |
| DHEAS:SIBG (African American) | -0.006 | 0.006 | PhenoAge | NA | 0.75 |
| DHEAS | -0.003 | 0.002 | GrimAge | 0.0005 | 0.75 |
| DHEAS:sex (women) | 0.006 | 0.004 | GrimAge | NA | 0.75 |
| DHEAS:SIBG (African American) | -0.002 | 0.004 | GrimAge | NA | 0.75 |
| DHEAS | -0.003 | 0.002 | Mean EAA | <0.0001 | 0.75 |
| DHEAS:sex (women) | 0.004 | 0.003 | Mean EAA | NA | 0.75 |
| DHEAS:SIBG (African American) | -0.002 | 0.003 | Mean EAA | NA | 0.75 |
| cortisol/DHEAS ratio | 0.182 | 0.218 | Horvath | 0.0002 | 0.75 |
| cortisol/DHEAS ratio:sex (women) | -0.135 | 0.270 | Horvath | NA | 0.75 |
| cortisol/DHEAS ratio:SIBG (African American) | 0.079 | 0.276 | Horvath | NA | 0.83 |
| cortisol/DHEAS ratio | 0.202 | 0.171 | Horvath2 | 0.004 | 0.75 |
| cortisol/DHEAS ratio:SIBG (African American) | 0.247 | 0.217 | Horvath2 | NA | 0.75 |
| cortisol/DHEAS ratio:sex (women) | -0.116 | 0.212 | Horvath2 | NA | 0.75 |
| cortisol/DHEAS ratio:SIBG (African American) | 0.444 | 0.248 | Hannum | NA | 0.75 |
| cortisol/DHEAS ratio | 0.096 | 0.196 | Hannum | 0.008 | 0.75 |
| cortisol/DHEAS ratio:sex (women) | 0.172 | 0.242 | Hannum | NA | 0.75 |
| cortisol/DHEAS ratio | 0.259 | 0.309 | PhenoAge | 0.004 | 0.75 |
| cortisol/DHEAS ratio:SIBG (African American) | 0.531 | 0.392 | PhenoAge | NA | 0.75 |
| cortisol/DHEAS ratio:sex (women) | -0.029 | 0.383 | PhenoAge | NA | 0.96 |
| cortisol/DHEAS ratio | 0.177 | 0.218 | GrimAge | 0.0002 | 0.75 |
| cortisol/DHEAS ratio:sex (women) | -0.131 | 0.270 | GrimAge | NA | 0.75 |
| cortisol/DHEAS ratio:SIBG (African American) | 0.082 | 0.277 | GrimAge | NA | 0.83 |
| cortisol/DHEAS ratio | 0.168 | 0.148 | Mean EAA | 0.004 | 0.75 |
| cortisol/DHEAS ratio:SIBG (African American) | 0.210 | 0.188 | Mean EAA | NA | 0.75 |
| cortisol/DHEAS ratio:sex (women) | -0.049 | 0.184 | Mean EAA | NA | 0.83 |
